# Resistant and susceptible responses in alfalfa (*Medicago sativa*) to bacterial stem blight caused by *Pseudomonas syringae pv*. *syringae*

**DOI:** 10.1371/journal.pone.0189781

**Published:** 2017-12-15

**Authors:** Lev G. Nemchinov, Jonathan Shao, Maya N. Lee, Olga A. Postnikova, Deborah A. Samac

**Affiliations:** 1 USDA-ARS, Molecular Plant Pathology Laboratory, Beltsville, Maryland, United States of America; 2 USDA-ARS, Plant Science Research Unit, St. Paul, Minnesota, United States of America; University of the West of England, UNITED KINGDOM

## Abstract

Bacterial stem blight caused by *Pseudomonas syringae* pv. *syringae* is a common disease of alfalfa (*Medicago sativa* L). Little is known about host-pathogen interactions and host defense mechanisms. Here, individual resistant and susceptible plants were selected from cultivars Maverick and ZG9830 and used for transcript profiling at 24 and 72 hours after inoculation (hai) with the isolate PssALF3. Bioinformatic analysis revealed a number of differentially expressed genes (DEGs) in resistant and susceptible genotypes. Although resistant plants from each cultivar produced a hypersensitive response, transcriptome analyses indicated that they respond differently at the molecular level. The number of DEGs was higher in resistant plants of ZG9830 at 24 hai than in Maverick, suggesting that ZG9830 plants had a more rapid effector triggered immune response. Unique up-regulated genes in resistant ZG9830 plants included genes encoding putative nematode resistance HSPRO2-like proteins, orthologs for the rice *Xa21* and soybean *Rpg1-b* resistance genes, and TIR-containing *R* genes lacking both NBS and LRR domains. The suite of *R* genes up-regulated in resistant Maverick plants had an over-representation of *R* genes in the CC-NBS-LRR family including two genes for atypical CC_R_ domains and a putative ortholog of the Arabidopsis *RPM1* gene. Resistance in both cultivars appears to be mediated primarily by WRKY family transcription factors and expression of genes involved in protein phosphorylation, regulation of transcription, defense response including synthesis of isoflavonoids, and oxidation-reduction processes. These results will further the identification of mechanisms involved in resistance to facilitate selection of parent populations and development of commercial varieties.

## Introduction

Alfalfa (*Medicago sativa* L.) is a key forage crop for dairy producers in the U.S. and in countries around the world. In addition, it is an important component of sustainable agricultural systems because of its high biomass yield, role in soil and water conservation, biological nitrogen fixation that improves soil fertility, interruption of pest and pathogens in crop rotations, and for providing wildlife habitat [1].

Plant pathogens and nematodes that infect alfalfa cause substantial losses in yield and quality of forage and reduce stand life. Largely because of the autotetraploid nature of alfalfa and severe inbreeding depression, alfalfa breeding is traditionally done by phenotypic recurrent selection of plant populations. Genetic mechanisms underlying disease resistance in alfalfa populations obtained by conventional methods remain essentially unknown. Understanding these processes would facilitate selection of resistant parent populations for breeding commercial varieties and increase the percentage of resistant plants in released varieties.

Bacterial stem blight of alfalfa, caused by *Pseudomonas syringae* pv. *syringae*, is common in the central and western U.S. and the disease occasionally occurs in eastern states. It has also been reported in Australia, Europe, and recently reported in western Iran [2]. There are two related phases of the disease, localized foliar necrosis (blight) and systemic vascular wilt. Infection of foliage results in water soaked lesions followed by chlorosis and necrosis of leaves. The bacterium penetrates host stems primarily at frost injury sites and forms water-soaked lesions that extend down the stem becoming amber with dried bacterial exudate that blackens with age. Plants with the disease are stunted, with spindly stems that are easily broken. Yield losses from the disease can be as high as 50% of the first harvest [3]. Disease losses in the first harvest are economically damaging because this harvest is typically the highest yielding with the best forage quality. With a changing climate, the possibility exists of expansion in the geographical range and impact of bacterial stem blight in the U.S. and globally.

*Pseudomonas syringae* is a Gram-negative bacterium that causes disease in practically every cultivated plant species [4]. It has been divided into approximately 50 pathovars (pv.), which are characterized by their host range. *P*. *syringae* pv. *syringae* is a very heterogeneous group whose members can cause disease collectively on over 200 plant species. The bacterium is capable of long distance aerial movement and there is strong evidence for its role in the global water cycle [5]. Long distance movement occurs when bacterial populations on plant surfaces are aerosolized and transported by air currents into the troposphere. They are then re-deposited on plants and in water systems in the form of snow and rainwater. The genetics of the interaction of *P*. *syringae* with plant hosts has been studied in both model plants and crop species [6, 7]. The resistance interaction follows the gene-for-gene interaction in which an effector protein from the bacterium is recognized by the resistance gene protein from the host to trigger a cascade of events leading to disease resistance. Initial defense is a hypersensitive response characterized by a rapid collapse/necrosis of infected cells. However, little is known about resistance in perennial plants such as alfalfa.

Previously, bacteria producing a fluorescent pigment were isolated from alfalfa with symptoms of bacterial stem blight from near Cheyenne, WY [8]. The strain ALF3 was identified as *P*. *syringae* pv. *syringae* (PssALF3) and a complete genome sequence was obtained [9]. It was found to be pathogenic on alfalfa, *Medicago truncatula* (barrel medic), bean seedpods, pear leaves, and beet seedlings but did not cause disease on bean leaves [8].

To date, there are no alfalfa cultivars selected for resistance to this disease and no known chemical control. Little is known about alfalfa-pathogen interactions: the means of bacterial pathogenicity, determinants of virulence, host defense mechanisms, and sources of resistance are absent in the literature. In this study, global transcriptome profiling of resistant and susceptible alfalfa plants was carried out using RNA-seq technology to identify alfalfa genes differentially expressed during infection. Key genes and processes involved in host resistance are proposed.

## Results

### Evaluation of phenotypic responses to bacterial stem blight in susceptible and resistant genotypes

**Fig 1:**
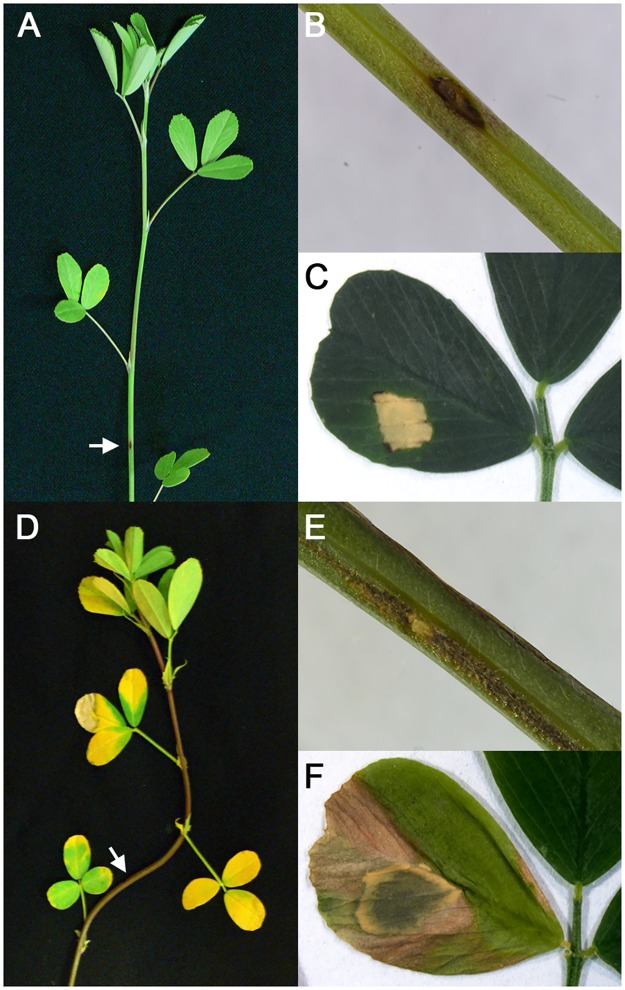
Symptoms of bacterial stem blight at 7 days after inoculation. **(A)** Response of resistant plant. **(B)** Stem of resistant plant at inoculation site. **(C)** Leaf of resistant plant at site of infiltration. **(D)** Response of susceptible plant. **(E)** Stem of susceptible plant at inoculation site. **(F)** Leaf of susceptible plant at site of infiltration. Arrows indicate point of inoculation.

Alfalfa cultivars Maverick and ZG9830 (Materials and methods) used in this work were found to have up to 59% of the plants resistant to bacterial stem blight (Samac, in preparation). Due to the obligate outcrossing genetics of alfalfa, resistant cultivars have a percentage of susceptible plants. To evaluate host resistance and susceptibility in individual alfalfa plants, their leaflets were infiltrated with PssALF3 and scored for disease symptoms. Either susceptible or resistant plants of both cultivars showed a necrotic response at 24 hours. However, for resistant plants the response did not change and in susceptible plants it progressed to water soaking followed by chlorosis and necrosis of cells beyond the site of infiltration (Fig 1). Seven days after inoculation resistant plants of both cultivars displayed browning of cells surrounding the site of inoculation on stems indicating a hypersensitive response (Fig 1). Susceptible plants showed water soaking and collapse at the site of inoculation and systemic chlorosis and necrosis of foliage. Determination of bacterial populations showed that plants scored as susceptible had a mean CFU (colony forming units) = log 8.2 while those scored as resistant had a mean CFU = log 6.4, evidence that susceptible plants with symptoms of disease promoted high bacterial populations. No symptoms and no CFU were obtained from mock-inoculated plants.

To uncover molecular mechanisms behind observed disease resistance or susceptibility and identify genes expressed by host plants in response to infection, RNA extracted from alfalfa plants was subjected to Illumina RNA sequencing. Individual resistant and susceptible plants were selected using disease symptoms that developed later in the course of infection, after RNA samples were collected at 24 and 72 hai.

### Transcriptome profiling of alfalfa plants in response to inoculation with PssALF3

The following hypotheses were tested: (1) host defense responses will be up-regulated in response to pathogen inoculation in resistant plants; and (2) host defense responses in susceptible plants will be suppressed and/or different from resistant plants upon inoculation.

A total of 6,176,469,338 pair-end reads were generated from 36 strand-specific cDNA libraries, averaging 171,568,593 reads per library (S1 Table). A total of 99.98% of all reads mapped to the reference CADL genome. Because cDNA libraries were generated using a poly(A) selection protocol, a minuscule number of bacterial transcripts mapped to the genome of PssALF3 (0.0017% of the total read counts) and were considered negligible. Therefore, the data obtained by RNA-seq were considered to be clean and sufficient for gene expression profiling.

#### Differentially expressed genes in ‘Maverick’ plants

Total counts of differentially expressed genes (DEGs) in Maverick plants at each time point (24 and 72 hai) are shown in S2 Table and quantitative estimates of DEGs in response to inoculation with PssALF3 are shown in Table 1. Four general observations can be made: (i) the number of DEGs at 72 hai is substantially larger than at 24 hai; (ii) the susceptible response generated more DEGs; (iii) the number of up-regulated DEGs in resistant plants is considerably higher at 72 hai than at 24 hai, and (iv) the proportion of DEGs up-regulated at 72 hai vs 24 hai in resistant plants is ~two-fold higher than in susceptible plants.

**Table 1:** Counts of differentially expressed genes in cv. Maverick.

Time point	Susceptible/Mock			Resistant/Mock		
	up	down	total	up	down	Total
**24 hrs**	1,851	554	2,405	962	114	1,076
**72 hrs**	2,098	985	3,083	2,079	583	2,662
**Unique**						
**24**	760	427	1,187	321	108	429
**72**	1,007	858	1,865	1,438	577	2,015
**common**	1,091	127	1,218	641	6	647

To further investigate this quantitative variability in expression we identified unique and common DEGs at both time points in susceptible and resistant interactions and between susceptible and resistant plants. Between 24 and 72 hai the number of unique DEGs increased in both interactions, especially in resistant plants (more than a four-fold increase). If the defense response in resistant Maverick plants is at its maximum at 72 hai, these unique DEGs, particularly those up-regulated, are likely to include genes implicated in resistance pathways, whereas common DEGs potentially include genes implicated in a general response to infection (S3 Table).

**Fig 2:**
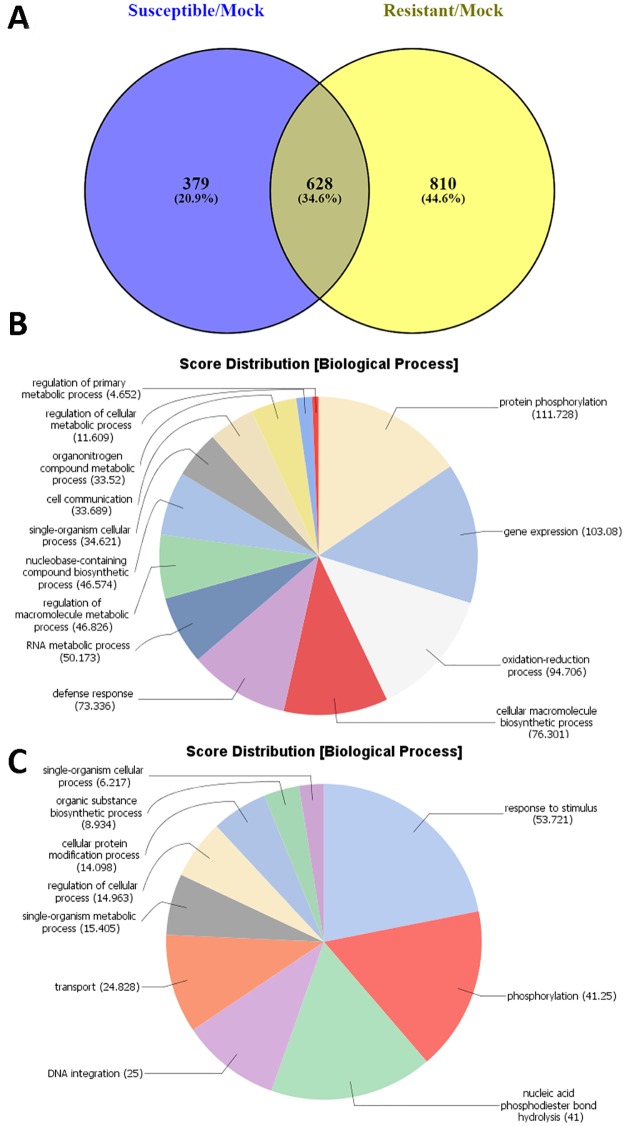
Differentially expressed genes (DEGs) in alfalfa plants from cultivar Maverick in response to inoculation with *P*. *syringae* pv. *syringae* ALF3. **(A)** Venn diagram depicting the unique and common up-regulated DEGs between susceptible and resistant plants at 72 hours after inoculation. **(B)** Functional profiling of the unique genes up-regulated genes in resistant plants (810 DEGs) using the Blast2Go tool. **(C)** Functional profiling of the unique genes up-regulated genes in susceptible plants (379 DEGs).

A comparison between the unique up-regulated genes at 72 hai in resistant/mock vs susceptible/mock interactions (1,438 DEGs vs 1,007 DEGs, respectively) (Table 1**)**, identified 810 genes up-regulated only in resistant plants (Fig 2A and S4 Table). Based on the BLASTX hits and gene annotations, at least 56 (6.9%) out of these unique up-regulated DEGs in resistant plants represent genes putatively encoding disease resistance proteins (blastx E-value cut-off ≤ 1e-10). Most of them (~44 DEGs) encode major classes of plant resistance (*R*) genes. Approximately forty-seven DEGs (5.8%) are putative transcription factors (TFs); among them highly-induced TFs of WRKY (20), MYB (8) and NAC (7) families that are implicated in stress responses and developmental programs. A large group of DEGs (~82 genes, 10.1%) putatively encodes receptor-like kinases (RLK), a superfamily of proteins involved in elicitor perception and *R* gene mediated responses [10]. One of these (Medtr0602s0020.1), a likely ortholog of *FLS2* (E-value = 0.0, identity 100%) encoding the receptor of flg22 (GeneBank accession XP_013441712), a component of bacterial flagellin and a potent elicitor of defense responses [11], was uniquely up-regulated at 72 hai in resistant plants. In addition, a substantial number of genes involved in isoflavonoid biosynthesis were found among 810 unique DEGs up-regulated in Maverick (S4 Table).

While the sets of unique DEGs up-regulated in resistant and susceptible plants of Maverick at 72 hai (810 and 379 DEGs, respectively) include a number of tentative *R* genes, most members of the TIR-NBS-LRR class, only resistant plants had differentially expressed *R* genes of the CC-NBS-LRR (CNL) class (nine *R* genes, E-value = 0 for all). Both TIR-NBS-LRR and CNL proteins are involved in pathogen recognition, although their sequence and signaling pathways are different (McHale et al., 2006). The N-terminal domain of *R* proteins (TIR or CC) is important for interaction with different proteins, including TFs [12] and can define specificity of pathogen recognition [13].

We attempted to identify orthologs of several well-known resistance genes, including *R* genes against *P*. *syringae* in the 810 unique genes up-regulated in Maverick. The genes included *RPM1* and *RPS2* from Arabidopsis, *Rpg1-b* from soybean, and an ortholog for a rice disease resistance gene *Xa21*, conferring resistance to *Xanthomonas oryzae* pv. *oryzae* [10, 14,15,16]. The respective sequences were used as queries against the entire CADL genome using standalone BLAST. Because of the conserved motifs in *R* genes, the search yielded numerous hits with different gene IDs in our DEG sets. When only sequences with the lowest E-value, higher bit scores and percent identities were considered, three candidates were predicted as potential orthologs of *RPM1*, *RPS2*, and *Xa21*: Medtr5g027900.1 (log2 fold change = 5.1, E-value = 1E-139, identity 31.1%, bit score = 446), Medtr4g073840.1 (log2 fold change = 2.1, E-value = 9E-38, percent identity = 26.1%, bit score = 155), and Medtr8g470400.1 (log2 fold change = 2.64, E-value = 4E-156, bit score = 491, identity = 37%), respectively. Low E-values, significant percent identities, and high expression levels indicate that *RPM1* and *Xa21* orthologs may be involved in the resistance response to PssALF3 in Maverick plants. No putative orthologs were identified for *Rpg1-b*.

Functional categorization using the Blast2Go tool [17] supported roles of DEGs in disease resistance. The 810 DEGs from resistant plants were predominately in five categories of the Gene Ontology (GO) domain Biological Process: ‘defense responses,’ ‘gene expression,’ ‘protein phosphorylation,’ ‘oxidation-reduction process,’ and ‘cellular macromolecule biosynthetic process’ (Fig 2B). The functional category ‘defense response’ was absent from the 379 unique DEGs up-regulated in susceptible plants (Fig 2C).

**Fig 3:**
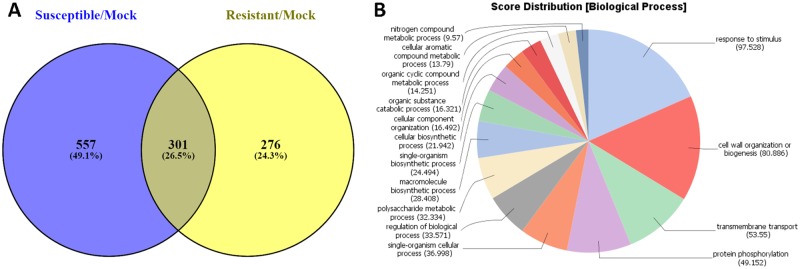
Down-regulated differentially expressed genes (DEGs) in alfalfa plants from cultivar Maverick. **(A)** Venn diagram depicting the unique and common down-regulated DEGs between susceptible and resistant plants at 72 hours after inoculation. **(B)** Functional profiling of the unique genes down-regulated in susceptible Maverick plants.

When unique down-regulated DEGs were compared between susceptible and resistant plants at 72 hai, 557 genes were found in the susceptible plants (Fig 3A). It is possible that down-regulation of these DEGs played a role in susceptibility to the infection, while common repressed genes between susceptible and resistant plants were not involved in the process. A list of the unique DEGs down-regulated in susceptible plants is shown in S5 Table. The unique down-regulated genes in susceptible plants were predominantly in the functional categories ‘response to stimulus,’ ‘cell wall organization and biogenesis,’ ‘transmembrane transport,’ and ‘protein phosphorylation’ (Fig 3B). GO term ‘response to stimulus’ include subcategories ‘response to biotic stimulus’ and ‘stress response.’ Down-regulation of the respective processes in susceptible plants may contribute to predisposition to infection.

#### Differentially expressed genes in ‘ZG9830’ plants

Total counts of DEGs found in ZG9830 plants in response to inoculation with PssALF3 are shown in S6 Table. While the susceptible response was similar to that observed in susceptible Maverick plants, the reaction of resistant plants to the infection was characterized by increased numbers of DEGs at 24 hai and decreased numbers of DEGs at 72 hai (Table 2). Most of the DEGs up-regulated in the resistant response at 24 hai were unique (61%). Resistant responses to infection appear to occur more rapidly in the selected ZG9830 plants than in the Maverick plants and expressed genes may be important in development of host resistance (S7 Table).

**Table 2:** Counts of differentially expressed genes in cv. ZG9830.

Time point	Susceptible/Mock			Resistant/Mock		
	up	down	total	up	down	total
**24 hrs**	2,106	522	2,628	2,731	830	3,561
**72 hrs**	2,609	999	3,608	1,307	193	1,500
**Unique**						
**24**	659	431	1,090	1,721	789	2,510
**72**	1,162	908	2,070	297	152	449
**common**	1,447	91	1,538	1,010	41	1,100

**Fig 4:**
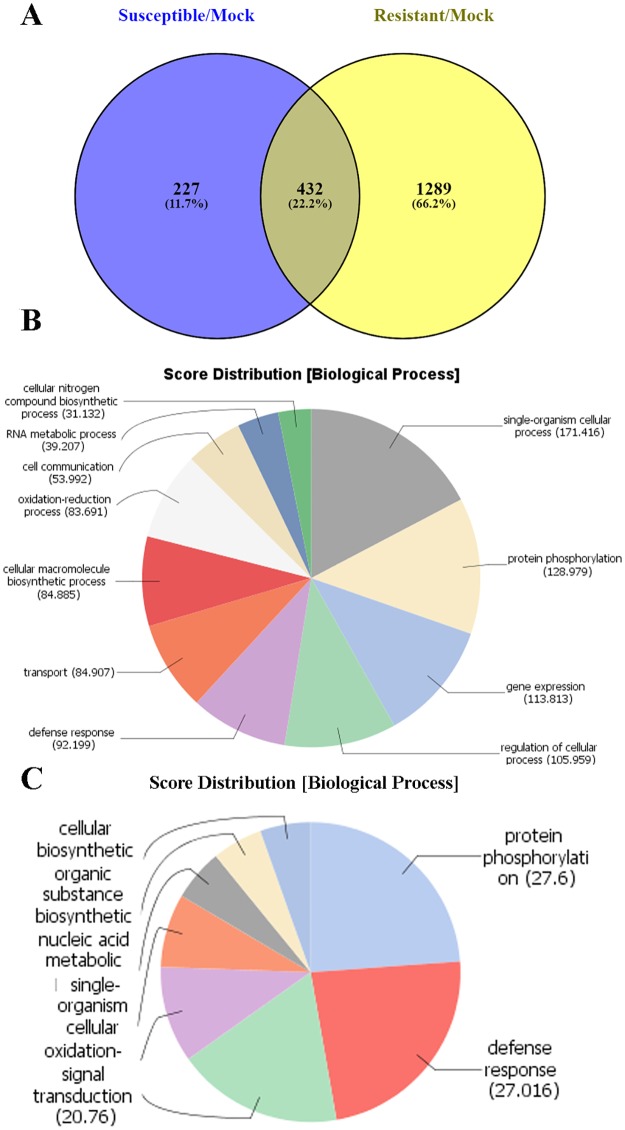
Differentially expressed genes (DEGs) in alfalfa plants from cultivar ZG9830 in response to inoculation with *P*. *syringae* pv. *syringae* ALF3. **(A)** Venn diagram depicting a number of unique and common up-regulated DEGs between susceptible and resistant plants at 24 hours after inoculation. **(B)** Functional categorization of the unique genes up-regulated in resistant plants. **(C)** Functional categorization of the unique DEGs induced in susceptible plants at 24 hai.

When we compared unique DEGs up-regulated at 24 hai in resistant and susceptible plants (1,721 and 659, respectively, in Table 2), 1,289 genes were induced only in resistant plants and 227 DEGs were induced only in susceptible plants (Fig 4A and S8 Table). At least 134 of the unique DEGs (10.3%) up-regulated in resistant plants at 24 hai represented genes putatively encoding *R* proteins. Of these, 83% (110 DEGs) belonged to the major classes of *R* genes, largely of the TIR-NBS-LRR class. Among up-regulated DEGs encoding putative *R* proteins in resistant plants, there were three nematode resistance HSPRO2-like proteins (E-values = 0, 0, and 1E-143), which were notably absent in the DEGs from susceptible plants. HSPRO2 has an imperfect LRR domain and lacks a nucleotide-binding site that is a characteristic feature of *R* proteins [18]. HSPRO2 was reported to interact with WRKY TFs positively regulating basal resistance in Arabidopsis against *P*. *syringae* pv. *tomato* [18]. It has also been previously suggested that Arabidopsis HSPRO2-like proteins could be implicated in a more general resistance process, not only nematode attack [19]. Also, TIR-containing genes lacking both NBS and LRR domains were up-regulated only in resistant ZG9830 plants. These TIR-unknown, or TX proteins, have been proposed to participate in plant basal defense responses [20]. Unlike the response in Maverick plants, *R* genes of the CNL type were up-regulated in both susceptible and resistant plants. Forty-nine DEGs (3.8%) encoded TFs, including members from the WRKY (13), MYB (8), AP2/ERF (6), and NAC (4) families. A substantial portion of the DEGs encoded receptor kinases (~140 genes, 10.8%). As in cv. Maverick, resistant plants of ZG9830 contained a group of up-regulated genes involved in the phenylpropanoid pathway leading to production of antimicrobial isoflavonoid phytoalexins (S8 Table) [21, 22].

The same orthologous *R* genes (*RPM1*, Medtr5g027900.1 and *RPS2*, Medtr4g073840.1) that were predicted in Maverick plants were found in the set of unique 1,289 DEGs up-regulated in ZG9830 plants (S8 Table) as well as several tentative *Xa21* orthologs. The best match to *Xa21* was Medtr5g025890.1, (log2 fold change = 4.14) with an E-value = 0 and 46% percent identity. We were also able to identify a putative ortholog of the soybean resistance gene *Rpg1-b* [14, 23]: Medtr8g038570.1 (E-value = 0, identity 49.5%, bit score 1027 and log2 fold change = 2.16). Thus, several potential orthologs of previously identified genes involved in resistance to bacterial pathogens were identified in resistant alfalfa plants and may participate in defense reaction against PssALF3.

Functional categorization of the unique genes up-regulated in resistant ZG9830 plants (1,289 DEGs) revealed at least eight prominent categories in the GO term Biological Process: ‘single-organism cellular process,’ ‘protein phosphorylation,’ ‘gene expression,’ ‘regulation of cellular process,’ ‘defense responses,’ ‘transport,’ ‘cellular macromolecule biosynthetic process,’ and ‘oxidation-reduction process’ (Fig 4B). Three of these categories were underrepresented in resistant Maverick plants: ‘single-organism cellular process,’ ‘transport,’ and ‘regulation of cellular process.’ This might indicate a more quantitative type of disease resistance in ZG9380 plants as compared to Maverick plants, with a greater number of genes contributing to resistance in the selected ZG9380 plants [24, 25]. Functional characterization of the unique DEGs up-regulated in susceptible ZG9380 plants at 24 hai (227 DEGs) revealed four prevalent GO terms: ‘protein phosphorylation,’ ‘defense response,’ ‘signal transduction,’ and ‘oxidation’ (Fig 4C). Most likely, up-regulated DEGs from these categories are part of the basal resistance pathways that are effectively overcome by the pathogen [26].

**Fig 5:**
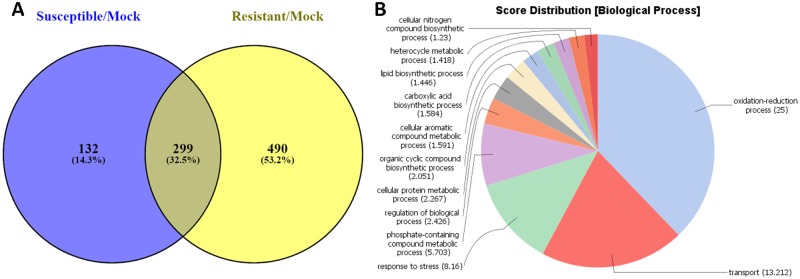
Down-regulated differentially expressed genes (DEGs) in alfalfa plants from cultivar ZG9830. **(A)** Venn diagram depicting a number of unique and common down-regulated DEGs between susceptible and resistant plants 24 hours after inoculation. **(B)** Functional categorization of the unique genes down-regulated genes in susceptible plants.

Comparison of the unique down-regulated DEGs between resistant and susceptible ZG9830 plants at 24 hai (789 vs 431 DEGs, respectively) identified 132 genes in the susceptible response (Fig 5A and S9 Table). As in the case with Maverick plants, these down-regulated genes could play a role in susceptibility to the infection, whereas the 299 down-regulated genes in common between susceptible and resistant plants may not be involved in the infection process. Functional profiling of the unique genes down-regulated genes in susceptible plants (132 DEGs) identified three predominant categories: ‘oxidation reduction,’ ‘transport,’ and ‘response to stress’ (Fig 5B). Genes in these categories play key roles during plant acclimation to stress [27, 28].

#### Comparison of the unique up-regulated DEGs from Maverick and ZG9830

**Fig 6:**
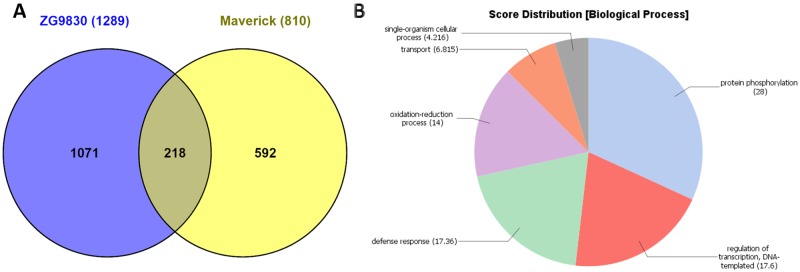
Comparison of differentially expressed genes (DEGs) in resistant plants from Maverick and ZG9830. **(A)** Venn diagram depicting the number of common DEGs between resistant plants. **(B)** Functional categorization of biological processes associated with resistance responses to *P*. *syringae* in plants from cv. Maverick and cv. ZG9830.

To determine if the same genes participate in resistance responses in both cultivars, we compared unique DEGs up-regulated in Maverick and ZG9830 at 72 hai (810 DEGs) and 24 hai (1,289 DEGs). A total of 218 DEGs were in common (Fig 6A and S10 Table). Functional annotation of biological processes associated with these DEGs identified four prevalent GO terms, accentuating their roles in both genotypes: ‘protein phosphorylation,’ ‘regulation of transcription, DNA-templated,’ ‘defense response,’ and ‘oxidation-reduction process’ (Fig 6B). Among these common up-regulated DEGs were 20 *R* genes, including four of the CNL class. A putative homolog of the gene encoding the nematode resistance HSPRO2 protein was up-regulated in resistant plants of both cultivars. Common TFs included eight WRKY, five MYB, and five members of other TF families. Therefore, it appears that WRKY and MYB TFs have a dominant role in regulating disease resistance networks against *P*. *syringae* in both cultivars [29].

#### Confirmation of the transcriptomic data by quantitative real-time PCR (qPCR)

qPCR was performed with 23 arbitrarily selected genes identified as differentially expressed based on analysis of the transcriptome (Table 3 and S11 Table). DEGs that were unique or common between the two cultivars were evaluated. qPCR data and the corresponding RNA-seq values were similar for all genes tested (Table 3). Importantly, up-regulation of genes encoding disease-resistance proteins was confirmed by qPCR.

**Table 3:** Confirmation of the transcriptomic data by quantitative real-time PCR.

Cultivar	Gene ID/Medtr ID	Description [Medicago truncatula]	Primers	qPCR	RNA-seq
				Log2-Fold Change	
Maverick	ID = g27133/Medtr0014s0220.1	PIF1-like helicase	LN460-461	-6.26	-6.6
Maverick	ID = g50748/Medtr2g079990.1	NAC transcription factor-like protein	LN464-465	4.61	4.43
Maverick	ID = g77527/Medtr5g065843.1	Transmembrane protein, putative	LN468-469	-8.63	-6.61
Maverick	ID = g80095/Medtr2g035150.1	Disease-resistance response protein	LN472-473	9.91	8.5
Maverick	ID = g85508/Medtr5g010640.1	Pathogenesis-related thaumatin family protein	LN474-475	7.64	6.65
Maverick	ID = g98659/Medtr1g067650.1	C2H2 type zinc finger transcription factor family protein	LN476-477	8.18	7.17
Maverick	ID = g124797/Medtr2g035150.1	Disease-resistance response protein	LN486-487	1.05	8.9
ZG9830	ID = g5392/Medtr2g026710.1	Nuclear transcription factor protein Y protein	LN452-453	-2.56	-4.08
ZG9830	ID = g20713/Medtr4g123990.1	ABC transporter B family protein	LN458-459	7.38	8.41
ZG9830	ID = g134174/Medtr3g467270.1	Salicylic acid carboxyl methyltransferase	LN492-493	-1.22	-4.03
ZG9830	ID = g8661/Medtr6g472230.1	Disease resistance protein (TIR-NBS-LRR class), putative	LN500-501	6.52	6.01
ZG9830	ID = g79942/Medtr6g472230.1	Disease resistance protein (TIR-NBS-LRR class), putative	LN502-503	5.11	5.82
ZG9830	ID = g91572/Medtr3g011390.1	NBS-LRR disease resistance protein	LN504-505	3.93	3.08
ZG9830	ID = g102799/Medtr5g037700.1	Disease resistance protein (TIR-NBS-LRR class)	LN506-507	3.49	2.28
ZG9830	ID = g107375/Medtr4g073840.1	NBS-LRR type disease resistance protein	LN508-509	1.96	2.22
ZG9830	ID = g60077/Medtr1g090680.1	LRR and NB-ARC domain disease resistance protein	LN466-467	-2.31	-3.37
ZG9830	ID = g79257/Medtr5g013440.1	Expansin-B1-like protein	LN470-471	-6	-6.6
ZG9830	ID = g130558/Medtr5g095840.1	Transmembrane protein, putative	LN490-491	-4.67	-5.4
ZG9830	ID = g139043/Medtr6g465530.1	Dehydration-responsive element-binding protein	LN494-495	-4.5	-5.28
ZG9830	ID = g110113/Medtr4g080777.1	Disease resistance protein (TIR-NBS-LRR class), putative	LN510-511	5.13	3.17
ZG9830	ID = g128367/Medtr8g020300.1	Disease resistance protein (TIR-NBS-LRR class)	LN512-513	3.63	2.28
ZG9830	ID = g136515/Medtr4g018940.1	Disease resistance family protein/LRR protein	LN514-515	3.87	2.71
ZG9830	ID = g150897/Medtr6g472230.1	Disease resistance protein (TIR-NBS-LRR class), putative	LN516-517	7.06	5.63

#### Detection of simple sequence repeats (SSRs)

Simple sequence repeats can provide valuable information on genetic polymorphism between resistance and susceptible plants. We restricted the analysis of SSRs to a few unique up-regulated genes, which presumably play key roles in resistance to PssALF3 in each cultivar. We focused on nine up-regulated *R* genes of the CNL class and the *Xa21* ortholog from Maverick plants and seven genes encoding TX and HSPRO-2-like proteins, and orthologs of the *Xa21* and *Rpg1-b* genes from ZG9830. Mining of SSRs, including hexamers with a minimum number of repeats two in the selected genes resulted in 1,207 and 758 SSRs in Maverick and ZG9830, respectively. Data are presented in S12 Table.

## Discussion

The interaction of *P*. *syringae* with plant hosts has been studied extensively and has informed much of the current understanding of the plant immune system [30, 31, 32, 33]. However, only a few studies on molecular interactions between *P*. *syringae* and different legume species have been described [15, 34, 35]. This is the first investigation of molecular responses of alfalfa to *P*. *syringae*. Here, we report that a highly pathogenic strain of *P*. *syringae* pv. *syringae* isolated from alfalfa caused transcriptional reprogramming of thousands of genes in susceptible and resistant genotypes of both cultivars used in this study. Although resistant plants from the two cultivars responded similarly to inoculation with a hypersensitive response at the site of inoculation, gene transcript profiling revealed that they respond differently at the molecular level (Tables 1 and 2) suggesting that resistance mechanisms are different between the two cultivars.

Two distinct but overlapping pathways of plant immune system have been previously defined: pattern-triggered immunity (PTI) and effector triggered immunity (ETI) [31, 32]. In PTI, conserved pathogen components, termed pathogen-associated molecular patterns (PAMPs) are recognized by host receptors, triggering a large transcriptional response. In susceptible plants, effectors produced by specialized pathogens can suppress PTI-based defenses and promote disease. In ETI, recognition of pathogen effectors by plant R proteins triggers the resistance response [31]. Up-regulated genes early in PTI and ETI are similar in Arabidopsis-*P*. *syringae* interactions but differ in magnitude with ETI having an earlier, stronger, and more extended response than PTI [36]. Similar sets of genes and patterns of gene expression occur in legumes responding to *P*. *syringae* with some exceptions. In soybean and *M*. *truncatula*, resistant plants have a strong and sustained up-regulation of genes in the phenylpropanoid pathway leading to production of isoflavonoid phytoalexins and there is a down-regulation of chloroplast related genes, which may favor production of reactive oxygen species involved in defense [35, 37]. Legumes also strongly up-regulate a group of pathogenesis-related proteins, named PR-10 proteins, with undefined functions in defense responses. While some of these host-pathogen responses were observed in our analysis of the alfalfa-PssALF3 interaction, many unique aspects were identified.

The observation of a hypersensitive response at the site of inoculation in resistant plants suggests that both cultivars utilize ETI, although temporally different. The transcriptomic responses of resistant ZG9830 plants indicate they may have a stronger and more rapid ETI than resistant Maverick plants, which had a later response to the infection with more differentially expressed genes at 72 hai compared to 24 hai (Table 1). In contrast, resistant plants from ZG9830 had more differentially expressed genes at 24 hai compared to 72 hai (Table 2). Whether resistance in ZG9830 is due to a quick and robust ETI response [38], a highly effective PTI-mediated signaling initiated early in host-pathogen interaction [31, 39], or a combination of ETI and PTI, is currently unclear. Although examples of strong PTI are rare, it can sometimes result in a hypersensitive response [38]. Data of expression of key resistance response genes from additional earlier and intervening time points are needed to confirm these observations.

Our analysis of DEGs in resistant ZG9830 plants identified a group of up-regulated genes with homology to known *R* genes that may be instrumental in resistance to PssALF3: four TIR-containing *R* genes lacking both NBS and LRR domains (TIR-unknown, or TX proteins [20], three up-regulated genes encoding nematode resistance HSPRO2-like proteins containing an imperfect LRR domain but no NBS [18], Medtr5g025890.1, encoding a putative ortholog for the rice *Xa21* gene, and Medtr8g038570.1, a proposed ortholog of the soybean resistance gene *Rpg1-b* (Table 4). In a scenario in which ZG9830 primarily utilizes ETI, the observed up-regulation of DEGs orthologous to the *Xa21* and *Rpg1-b* resistance genes could imply mechanisms similar to *Xa21* signaling in rice, in which the *R* protein acts as a pathogen recognition receptor for the avrXa21 effector from *Xanthomonas oryzae* pv. *oryza* [10, 40] or to *Rpg1-b* signaling in soybean, where it mediates detection of the *P*. *syringae* pv. *glycinea* effector protein AvrB [15]. The up-regulation of genes encoding TX and HSPRO2-like proteins, that were proposed to participate in or positively regulate plant basal defense responses [18, 20] may signify the importance of PTI in resistant ZG9830 plants. The large number of functional categories of induced DEGs in resistant ZG9830 plants is indicative of a diverse type of resistance response that might be driven not by a single gene but rather by a contribution of multiple individual genes, each playing a role in the outcome [24, 25].

**Table 4:** Candidate genes involved in the resistance response against PssALF3 in two alfalfa cultivars.

cv. Maverick			cv. ZG9830		
Medtr ID	Log2 Fold Change	Description	Medtr ID	Log2 Fold Change	Description
**Medtr5g027860.1**	5.79	CC-NBS-LRR	Medtr2g079950.1	7.90	TIR-unknown (TX)
Medtr5g027900.1*	5.15	CC-NBS-LRR	Medtr2g079950.1	6.87	TIR-unknown (TX)
Medtr5g027900.1*	4.70	CC-NBS-LRR	Medtr3g070230.1	3.59	HSPRO2-like
**Medtr8g079520.1**	3.07	CC_**R**_-NBS-LRR	Medtr0277s0020.3	2.76	TIR-unknown (TX)
**Medtr2g038510.1**	2.74	CC-NBS-LRR	Medtr5g092220.1	2.66	TIR-unknown (TX)
**Medtr5g018120.1**	2.61	CC-NBS-LRR	Medtr5g082150.1	2.42	HSPRO2-like
**Medtr2g038510.1**	2.52	CC-NBS-LRR	Medtr5g082150.1	2.08	HSPRO2-like
**Medtr5g027910.1**	2.48	CC-NBS-LRR	Medtr5g025890.1	4.14	LRR-RLK
**Medtr5g018210.1**	2.29	CC_**R**_-NBS-LRR	Medtr8g038570.1	2.16	NBS-LRR
**Medtr8g470400.1**	2.64	LRR-RLK			

*unique alfalfa genes that map to the same gene in the *M*. *truncatula* genome

In Maverick plants, the transcriptome analysis suggests that an early and relatively weak PTI does not contain the pathogen in either susceptible or resistant plants and is overpowered by secreted pathogen effectors [32]. In this scenario, one of the nine genes encoding a group of CNL proteins up-regulated at 72 hai in resistant plants could potentially be responsible for recognition of bacterial effectors and triggering an ETI response that contains the pathogen. Importantly, two of the nine CNL class genes encode atypical CC_R_ domains [41], which alone are sufficient for induction of defense responses in Arabidopsis (Collier *et al*., 2011) [42], are strongly up-regulated in resistant Maverick plants at 72 hai (Table 4). The CC_R_ domain in Medtr8g079520.1 and Medtr5g018210.1 resembles The CC_R_ domain in the Arabidopsis RPW8 protein (locus RESISTANCE TO POWDERY MILDEW8) that mediates broad-spectrum mildew resistance [43]. Putative orthologs for the Arabidopsis *RPM1* gene conferring resistance to *P*. *syringae* and for rice *Xa21* gene, conferring resistance to *Xanthomonas oryzae* pv. *oryzae* [16], could also play a role in the resistance response in Maverick plants, although errors in identification of homologous genes remain a possibility due to highly conserved features in predicted candidate genes. Functional categorization of the unique DEGs up-regulated at 72 hai showed that susceptible Maverick plants lacked a GO category ‘defense response,’ whereas this term was one of the most prevalent in resistant plants.

A significant number of induced *R* genes found in both cultivars suggests possible roles in the PssALF3-alfalfa interaction. Regulation of the expression of *R* genes has been found to occur through transcriptional and post-transcriptional mechanisms to maintain the proper level to sufficiently activate resistance without having negative effects on fitness or plant growth [44, 45]. Transcripts of many *R* genes have been shown to accumulate in response to pathogen attack as well as abiotic stresses [46]. Defense-associated up-regulation of *R* genes contributes to PTI and ETI, may enhance potency of pathogen recognition, and has been observed in other plant species [44, 47].

Functional characterization of DEGs found in common in resistant plants of the two cultivars showed that four major biological processes predominated: ‘protein phosphorylation,’ ‘regulation of transcription,’ ‘defense response,’ and ‘oxidation-reduction.’ A substantial portion of the DEGs was associated with each of these categories (S9 Table). These four processes appear critical to plant defense signaling and activity against PssALF3 in alfalfa. Among differentially expressed TFs in the category ‘regulation of transcription,’ WRKY TFs appear to play a critical role in regulation of defense mechanisms against *P*. *syringae* in both cultivars. WRKY family TFs are involved in regulation of key constituents of the plant immune system including PTI, ETI, and systemic acquired resistance [48]. Redox signaling and production of reactive oxygen species also appear to be at the core of defense response in both cultivars, particularly in resistant Maverick plants (Fig 6A and 6B).

## Conclusions

**Fig 7:**
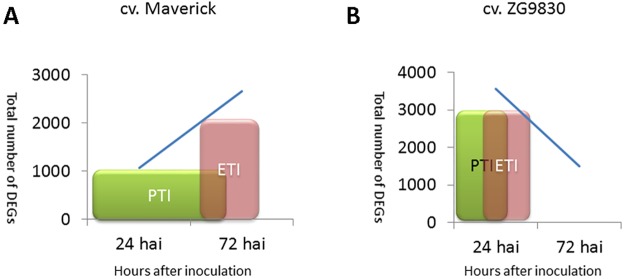
Hypothetical mechanisms of resistance to PssALF3. (**A**) Resistant plants of cv. Maverick. **(B)** Resistant plants of cv. ZG9830. PTI, pathogen-associated molecular patterns (PAMP)-triggered immunity. ETI, effector-triggered immunity [31].

In summary, evaluation of resistant and susceptible responses to *P*. *syringae* pv. *syringae* showed that timing of the resistance response and candidate genes involved in resistance differed between the two cultivars. The development of a hypersensitive response and suppression of bacterial populations in plants categorized as resistant suggests the plants rely on ETI, particularly in the plants selected from cultivar ZG9830. The response of resistant plants from cultivar ZG9830 to the pathogen occurred earlier and may be coordinated by NBS-LRR, TX, and HSPRO2-like *R* proteins. Resistant plants from the cultivar Maverick responded later and *R* genes in the CNL class may be important in triggering resistance. Based on our interpretation of the data we propose a schematic representation of defense signaling in response to PssALF3 in plants of both alfalfa cultivars (Fig 7). Other interpretations of the data using different comparisons are possible and may reveal additional outcomes and conclusions. Additionally, our focus on selected components of the plant immune system may not reflect the complexity of this adaptive host-pathogen interaction. Notwithstanding, this study provides the first comprehensive study of the transcriptional response of alfalfa plants to *P*. *syringae* pv. *syringae*, an agriculturally important bacterial pathogen of alfalfa. Our results suggest that utilization of ZG9830 as a source of resistance for developing bacterial blight resistant cultivars may be more productive than use of Maverick as a source germplasm.

## Methods

### Plant material and bacterial inoculation

Resistant and susceptible alfalfa plants for transcript profiling were selected from cultivars Maverick [49] and ZG9830 [50]. To select plants, scarified seed were planted in a peat-based potting mix (Sungro LC8, SunGrow Horticultural Distribution, Agawam, MA) and grown for 5 weeks in the greenhouse at approximately 25°C with a 16 h photoperiod until they had formed four true leaves. The bacterial inoculum was prepared by culturing PssALF3 on King’s B agar for 2 days at 25°C. Cells were harvested in sterile distilled water and adjusted to an optical density OD_600_ = 0.1, approximately 1.5 x 10^8^ colony forming units (CFU)/ml. The third internode above the cotyledons was wounded at a single site using a 22-gauge needle and the wound swabbed using a sponge moistened with the bacterial inoculum. After symptoms appeared, diseased material was removed and plants were allowed to re-grow. Vegetative clones of selected plants were made and re-tested for disease symptoms by stem inoculation, leaflet infiltration and determination of bacterial populations. Leaflets were infiltrated with the bacterial inoculum on the abaxial side using a needless syringe. To determine bacterial populations, inoculated internodes were scored for disease symptoms at 7 d after inoculation then excised using a sterile razor blade. These stem sections were placed in sterile water for 1 h and the resulting bacterial suspension used to make serial dilutions. Bacteria were cultured King’s B agar and CFU determined after 2 d incubation at 25°C. For transcript profiling, plants were inoculated at three sites per internode by wounding with a 22-gauge needle and swabbing with a suspension of PssALF3 at OD_600_ = 0.1. Mock-inoculated plants were wounded with a 22-gauge needle and swabbed with sterile water. Internodes were excised at 24 and 72 h after inoculation (hai), frozen immediately in liquid nitrogen, then stored at -80°C until used for RNA extraction.

### RNA extraction and RNA-seq

Three resistant, three susceptible, and three mock-inoculated plants from cvs. Maverick and ZG9830 were chosen for transcript profiling at each time point. RNA was extracted using the RNAeasy Plant Mini Kit (Qiagen) and treated with DNase to remove any remaining DNA from total RNA samples. Purity and quantity of the samples was checked with a NanoDrop spectrophotometer (Thermo Scientific, USA) and Agilent 2100 BioAnalyzer. After total RNA was extracted from plants at 24 and 72 hai, they were maintained in the greenhouse to observe symptoms and classify plants as susceptible or resistant. RNA sequencing was performed by the Center for Computational Genomics, Next Generation Sequencing Center, Johns Hopkins University. cDNA libraries were generated using a poly (A) selection method and TruSeq RNA Library Preparation kit (Illumina, Inc.) Paired-end reads (2 x 150 bp) were generated using the Illumina HiSeq 2500 sequencing system. Four samples were pooled into each individual lane.

### Read mapping, quantification, and functional analysis

The whole genome sequence of cultivated alfalfa at the diploid level (CADL, 2*n* = 2*x* = 16; CADL_HM342.v0.95P) was obtained from the Medicago HapMap project (http://www.medicagohapmap.org/home/view) and putative gene predictions were made using the AUGUSTUS (2.7) (http://augustus.gobics.de/) gene prediction tool. Gene IDs used in this manuscript were extracted from the AUGUSTUS output. The strand-specific paired-end reads were mapped onto the CADL gene predictions using Bowtie aligner version 2.2.9 (http://bowtie-bio.sourceforge.net/index.shtml). The paired-end reads were mapped using the –nofw and –norc parameters to best capture strand specific sequencing. Gene annotation was based on BLASTX hits with the *M*. *truncatula* genome database downloaded from the NCBI Genbank (https://www.ncbi.nlm.nih.gov/). The *M*. *truncatula* IDs (Medtr) were assigned using the BLASTX program with Mt4.0, which can be downloaded from the *Medicago truncatula* Genome Database (http://www.medicagogenome.org/home). The DESeq 2 package from Bioconductor [51] was used to estimate sample quality and expression level of the genes. The raw counts obtained from the Bowtie alignments were normalized using the DESeq 2 program that calculated the size factors of each library based on the raw counts. The size factors were calculated by dividing each column of raw counts by the geometric mean of the rows between comparisons. The median of these ratios was used as the size factor for the column. Genes with fold change more than 2, false discovery rate (FDR) less than 0.05, and number of mapped reads more than 50 were counted as a differentially expressed. The Blast2GO tool was used for functional categorization of differentially expressed genes [17].

The distribution scores in the Gene Ontology (GO) charts represent the sum of sequences directly or indirectly associated to a given GO category weighted by the distance of the category to the term of "direct annotation". It was computed by the software according to the formula

$$score=\sum_{GOs}seq\;\text{x}\;\alpha^{dist}$$

where *seq* is the number of different sequences annotated in a child GO term and *dist* is the distance to the node of the child term (https://biobam.atlassian.net/wiki/display/BFCD/Gene+Ontology+Graph+Visualization).

### Verification of transcriptome data

Quantitative real-time PCR (qPCR) was performed with arbitrarily selected genes to confirm transcriptomic data. Primers were designed using the online Realtime PCR tool (Integrated DNA Technologies Inc., San Diego, USA; https://www.idtdna.com/scitools/Applications/RealTimePCR/) and alfalfa sequences generated in this work. cDNA for qPCR analyses was made using the SuperScript^™^ III First-Strand Synthesis System with oligo d(T) (ThermoFisher Scientific) and the same RNA samples that were used for RNA sequencing. Amplification was conducted with a Rotor Gene Q real time PCR cycler (Qiagen) using the Rotor-Gene SYBR^®^ Green PCR kit (Qiagen) with three biological replicates using the following parameters: 95°C for 10 min (one cycle), 95°C for 10 s and 60°C for 45 s (40 cycles). The Delta Delta C(T) method (2^−ΔΔ***C***^_T_) was used for analysis of relative expression [52]. To obtain a final ratio for any given gene, an average and a standard deviation for all biological replicates was calculated. The reference gene in all qPCR experiments was NP_001237047, a gene of unknown function with little variation in expression levels [53].

### Detection of simple sequence repeats (SSR) markers

Identification of di-, tri-, tetra-, penta-, and hexanucleotide SSR loci, with a minimum repeat numbers of two was performed using the Simple Sequence Repeat Identification Tool (SSRIT, http://archive.gramene.org/db/markers/ssrtool).

## Supporting information

S1 Table RNA-seq metrics. (XLSX)

S2 Table Total counts of differentially expressed genes (DEGs) in resistant and susceptible plants of Maverick at each time point (24 and 72 hours after inoculation). [Maverick-resistant 24 hrs: 1076 DEGs; Maverick-resistant 72 hrs: 2,662 DEGs; Maverick-susceptible, 24 hrs: 2,405; Maverick-susceptible, 72 hrs: 3,083].
(XLSX)

S3 Table Total counts of unique differentially expressed genes (DEGs) in Maverick plants at each time point (24 and 72 hours after inoculation). [Maverick-resistant 24 hrs: 429 DEGs; Maverick-resistant 72 hrs: 2,015 DEGs; Maverick-susceptible, 24 hrs: 1,187 DEGs; Maverick-susceptible, 72 hrs: 1.865 DEGs].
(XLSX)

S4 Table Unique differentially expressed genes (DEGs) up-regulated only in resistant plants of Maverick at 72 hours after inoculation (810 DEGs). (XLSX)

S5 Table Unique differentially expressed genes (DEGs) down-regulated in susceptible Maverick plants at 72 hours after inoculation (577 DEGs). (XLSX)

S6 Table Total counts of differentially expressed genes (DEGs) in resistant and susceptible plants of ZG9830 at each time point (24 and 72 hours after inoculation). [ZG9830-resistant 24 hrs: 3.561 DEGs; ZG9830-resistant 72 hrs: 1,500 DEGs; ZG9830-susceptible, 24 hrs. 2,628; ZG9830-susceptible, 72 hrs: 3,608].
(XLSX)

S7 Table Total counts of unique down-regulated genes (DEGs) in ZG9830 plants at each time point (24 and 72 hours after inoculation). [ZG9830-resistant 24 hrs: 2,461 DEGs; ZG9830-resistant 72 hrs: 400 DEGs; ZG9830-susceptible, 24 hrs: 1,090 DEGs; ZG9830-susceptible, 72 hrs: 2,070 DEGs].
(XLSX)

S8 Table Unique down-regulated genes (DEGs) up-regulated only in resistant ZG9830 plants at 24 hours after inoculation (1,289 DEGs). (XLSX)

S9 Table Unique down-regulated genes (DEGs) in susceptible ZG9830 plants at 24 hours after inoculation (132 DEGs). (XLSX)

S10 Table Common up-regulated DEGs found in Maverick plants at 72 hours after inoculation and ZG9830 plants at 24 hours after inoculation (218 DEGs). (XLSX)

S11 Table Primers used for quantitative real-time PCR. (XLSX)

S12 Table SSRs found in resistance gene candidates of both cultivars. (XLSX)
